# Clinical trials of bispecific antibody therapy for colorectal cancer: advanced and next steps

**DOI:** 10.3389/fonc.2026.1758251

**Published:** 2026-02-11

**Authors:** Wen Shao, Yuhang Liu, Lina Huang, Sihan Lu, Yixiang Zhai, Yue Xiong, Nuojun Chen, Pengcheng Ye, Qijun Lv

**Affiliations:** 1 Department of Clinical School and Affiliated Hospital, North Sichuan Medical College, Nanchong, Sichuan, China; 2 Department of Medical Imaging, North Sichuan Medical College, Nanchong, Sichuan, China; 3 The People's Hospital of Gaozhou, Maoming, Guangdong, China; 4 Department of Nursing, North Sichuan Medical College, Nanchong, Sichuan, China

**Keywords:** bispecific antibodies, clinical trial landscape analysis, clinical trials, colorectal cancer, immunotherapy, target combinations

## Abstract

**Background:**

Colorectal cancer (CRC) is a malignant tumor with a high incidence and mortality rate worldwide. The existing treatment methods have limitations in terms of efficacy or applicable population. Bispecific antibodies (BsAbs) can simultaneously target two different antigens and are expected to overcome tumor immune escape, providing a new strategy for the treatment of CRC.

**Method:**

This study systematically retrieved clinical trial registration platforms such as Trialtrove and ClinicalTrials.gov up to July and November 2025, and collected trial data on the treatment of CRC with BsAbs. Descriptive analyses were conducted on key indicators such as the stage distribution, primary endpoints, funding types, global distribution, and target combinations of the trials by establishing clear inclusion and exclusion criteria.

**Result:**

A total of 192 clinical trials were included. Since 2018, the number of related trials has significantly increased, and the trial phase has shifted from mainly Phase I in the early stage to a substantial growth in Phase II and Phase III trials after 2023-2024. The primary endpoints of the trial were highly concentrated on safety assessment (such as safety/tolerability, adverse events). The industrial sector is the main funder (68.3%), and the number of trials conducted in China (n=125) ranks first in the world. The target combinations are most commonly PD-1/CTLA-4 and PD-1/VEGF, and studies on novel combinations such as EGFR/cMET are also on the rise. Efficacy data from key trials (e.g., Cadonilimab, Amivantamab) demonstrate encouraging response rates in both locally advanced and metastatic settings, particularly in MSS/pMMR populations.

**Conclusion:**

The clinical research and development activities of BsAbs in the field of CRC treatment are becoming increasingly active and mature. Currently, the focus is on establishing a safety profile. Dual-target blocking based on PD-1 and strategies targeting EGFR/cMET are the current main research and development directions. In contrast to resource-intensive CAR-T or payload-driven ADCs, BsAbs provide a ready-to-use therapeutic format that simultaneously engages two antigens, offering distinct practical and mechanistic benefits. In the future, it is necessary to further optimize the design of BsAbs, explore combination therapies and identify predictive biomarkers to promote its clinical transformation and improve the prognosis of CRC patients.

## 1 Introduction

Colorectal cancer (CRC) ranks among the most prevalent and lethal malignancies worldwide, posing a substantial public health challenge. It is the third most commonly diagnosed cancer and the second leading cause of cancer-related deaths globally (1). Epidemiological data reveal persistent high incidence and mortality rates, with notable geographic and demographic disparities. For instance, Asia accounted for 50.2% of global colorectal cancer cases in 2022, with incidence and mortality projected to rise significantly by 2050, particularly in low Human Development Index countries (2). Similarly, racial and ethnic disparities are evident in the United States, where African American populations exhibit higher incidence and mortality rates compared to other groups (3). Current CRC treatment modalities encompass surgery, chemotherapy, radiotherapy, targeted therapy, and immunotherapy. While these approaches have achieved varying degrees of success, significant limitations persist. Chemotherapy remains a cornerstone of treatment but is often hindered by chemoresistance and debilitating side effects (4, 5). Targeted therapies against epidermal growth factor receptor (EGFR) and vascular endothelial growth factor (VEGF) pathways have improved outcomes in select patient subsets; however, primary and acquired resistance mechanisms, such as mutations in RAS and BRAF genes, limit their long-term effectiveness (6, 7). Immunotherapy, particularly immune checkpoint inhibitors (ICIs), has revolutionized cancer treatment but demonstrates limited efficacy in CRC, largely confined to microsatellite instability-high (MSI-H) or mismatch repair-deficient (dMMR) tumors, which constitute a minority of cases (8, 9). The majority of CRC patients with microsatellite stable (MSS) disease do not benefit from ICIs due to an immunosuppressive tumor microenvironment and immune evasion mechanisms.

Given these challenges, there is a compelling rationale to explore novel immunotherapeutic modalities that can enhance antitumor immune responses with improved specificity and reduced toxicity. Among these, bispecific antibodies (BsAbs) have emerged as a promising class of agents that can simultaneously engage two distinct antigens, thereby redirecting immune effector cells to tumor cells and overcoming immune escape (10). BsAbs can be engineered to target tumor-associated antigens (TAAs) and immune cell receptors, such as CD3 on T cells, facilitating targeted cytotoxicity independent of major histocompatibility complex (MHC) restrictions (11). This dual specificity offers the potential to enhance treatment precision and efficacy while mitigating off-target effects.

Recent years have witnessed a growing number of clinical trials investigating BsAbs in CRC, targeting various antigens including carcinoembryonic antigen (CEA), epithelial cell adhesion molecule (EpCAM), guanylyl cyclase C (GUCY2C), and others (12–14). These studies have demonstrated encouraging antitumor activity and manageable safety profiles, highlighting the versatility of BsAbs in addressing tumor heterogeneity and resistance. Moreover, advances in antibody engineering have enabled the development of novel formats such as trispecific antibodies and bispecific antibody-drug conjugates (ADCs), further expanding therapeutic options (15–17). Importantly, combination strategies incorporating BsAbs with immune checkpoint blockade or chemotherapy have shown synergistic effects, suggesting avenues to overcome intrinsic resistance mechanisms (11, 18). Despite these promising developments, challenges remain in optimizing BsAb design, identifying suitable target antigens with high tumor specificity, and managing immune-related adverse events such as cytokine release syndrome (11). Additionally, the complex tumor microenvironment in CRC, characterized by immunosuppressive cells and signaling pathways, necessitates integrated approaches to enhance immune infiltration and activation (19). The heterogeneity of CRC at the molecular and cellular levels also underscores the need for personalized therapeutic strategies guided by biomarker profiling (7).

In this context, a systematic review and analysis of clinical trials employing bispecific antibodies in CRC is timely and critical. This study pioneeringly amalgamates data from worldwide clinical trial registration platforms to furnish a comprehensive descriptive analysis of the overarching framework, phase distribution, and status of drug development in current trials, thereby providing insights for the macro-level enhancement of future clinical trial designs.

## 2 Methods

**Figure 1:**
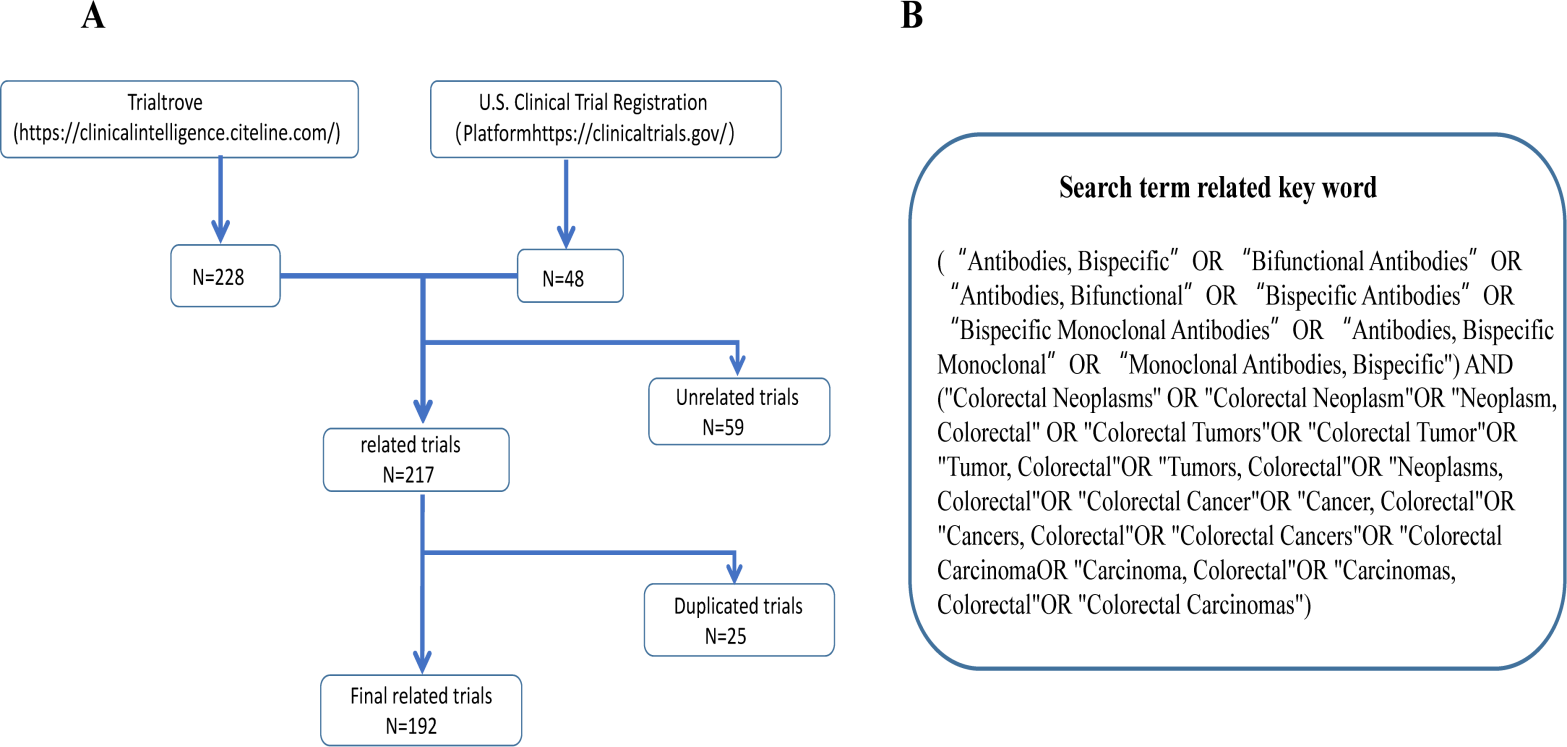
Clinical trial sources and inclusion and exclusion flowchart. **(A)** Clinical trial inclusion and exclusion flowchart; **(B)** search term related keyword.

We performed a systematic search of Trialtrove (https://clinicalintelligence.citeline.com/) on July 12, 2025. The initial search strategy employed the following query: (Disease is Oncology: Colorectal) AND (Drug Type is Biological > Protein > Antibody > Bispecific antibody). To maximize search comprehensiveness and minimize the risk of omission, a supplementary search was performed on November 25, 2025, across the primary registries endorsed by the International Clinical Trials Registry Platform (ICTRP), such as ClinicalTrials.gov. The same key concepts (colorectal cancer, bispecific antibodies) were applied to ensure consistency. The analysis of the identified trial records focused on the following key metrics: trial phase, recruitment status, primary endpoints, drug targets, and sponsor types along with their geographical distribution. Two investigators independently extracted and validated source data to ensure conclusion accuracy, according to the following procedure. This included processing unstructured data, resolving missing information, and mitigating classification bias, implementing rigorous quality control. Residual limitations do not compromise the study’s overall trends(**Figure 1**).

First, to avoid duplication of data, the studies that passed the initial screening were scrutinized for duplicate records. This involved comparing key trial identifiers, such as the clinical trial registration number and core study design elements. Duplicate entries were identified and merged or removed to ensure each trial was represented only once in the analysis.

Subsequently, the initial screening was performed based on the study title, design, and medical condition. Studies were included if they explicitly involved interventions with bispecific antibodies for colorectal cancer. The exclusion criteria were defined as follows: studies with a primary focus on diagnostics, those with a recruitment status of ‘withdrawn’, trials not related to colorectal cancer, or those not involving bispecific antibody therapy were excluded.

Finally, to maximize the comprehensiveness of our dataset, we also included trials focusing on solid tumors that explicitly listed colorectal cancer among the participant cohorts or cancer types studied, provided they met all other inclusion criteria. This approach aimed to capture all potentially relevant bispecific antibody trials applicable to the colorectal cancer population. To evaluate the geographic equity of trial participation, we further classified trial countries as low- or middle-income if their per-capita gross national income (GNI) was ≤ US $4,465 according to the World Bank 2023 threshold. Data extraction and management for the final included studies was performed using Microsoft Excel. The world map and bubble were produced using Tableau Software (v2019.4.4), and all other graphics were created using Origin 2021.

## 3 Results

### 3.1 Trial phases and temporal characteristics

**Figure 2:**
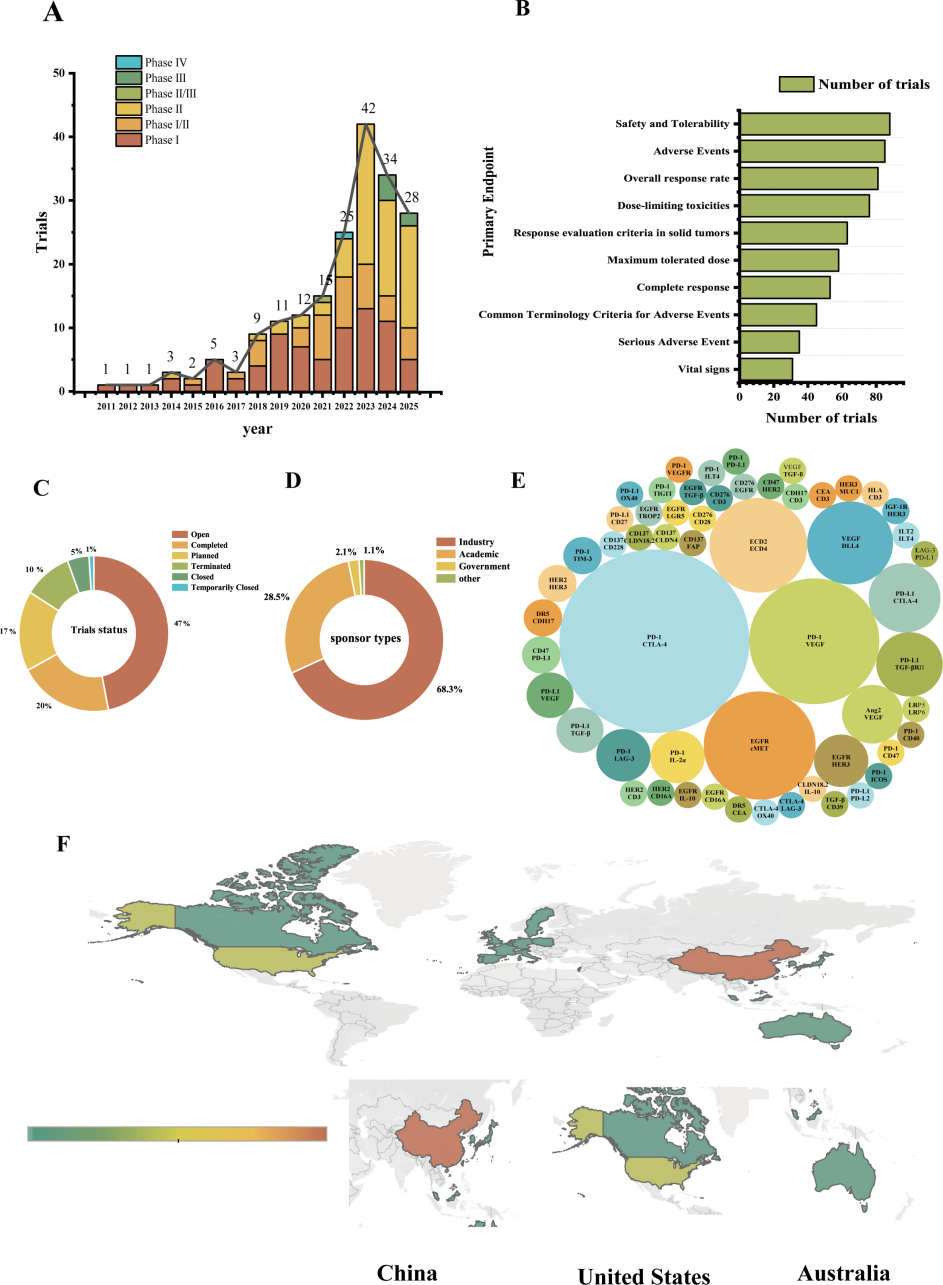
Comprehensive overview of multidimensional data analysis in clinical trials. **(A)** Distribution by year and trial phase. **(B)** Primary endpoints evaluated in clinical trials. **(C)** Status distribution of clinical trials. **(D)** Types of sponsors for clinical trials. **(E)** Drug target classes assessed in bispecific antibody clinical trials. **(F)** Global distribution of clinical trials, with darker red indicating higher registration number.

Analysis of clinical trial data from 2011 to 2025 is expected to reveal a marked expansion in bispecific antibody development for colorectal cancer, with a total of 192 trials initiated(**Figure 2A**). Activity was limited in the early years (2011–2017), but followed by a substantial increase from 2018 onward. Notably, clinical trials have undergone a fundamental shift: Phase I trials dominated the initial growth phase, but a notable surge in Phase II trials began in 2023 (n=22), becoming the most active phase in 2024-2025. Furthermore, the recent emergence of Phase III trials (starting in 2024, n=6) signals that the most advanced candidates are now entering large-scale, confirmatory studies.

### 3.2 Analysis of primary endpoints in clinical trials

Analysis of primary endpoints demonstrates a predominant focus on safety assessment in bispecific antibody trials for colorectal cancer(**Figure 2B**). The most frequent endpoints are “Safety and Tolerability” (n=88) and “Adverse Events” (n=85), followed by key safety metrics such as “dose-limiting toxicities” (n=76) and “maximum tolerated dose” (n=58). While efficacy endpoints like “overall response rate” (n=81) are also prominent, the consistent prioritization of safety parameters underscores the critical need to establish a tolerable therapeutic profile during clinical development of these novel agents.

### 3.3 Trial status and sponsorship landscape

Analysis of trial status across the 192 studies indicates a highly active and evolving field(**Figure 2C**). Nearly half of the trials (47%) are currently open for participant recruitment, while a significant proportion (17%) are in the planning stage. Completed trials account for 20% of the total. A non-negligible proportion of trials were terminated, accounting for 10% (n=20) of the total. Analysis of sponsorship demonstrates that industry is the predominant sponsor; responsible for approximately 68.3% of trials(**Figure 2D**). Academic institutions represent the second largest sponsor category (28.5%), while government and other entities play a minor role (2.1% and 1.1%, respectively).

### 3.4 Analysis of global distribution and international collaboration

The global distribution of clinical trials for bispecific antibodies in colorectal cancer demonstrates extensive international collaboration(**Figure 2F**). The largest and most diverse study (NCT06750094) shows participation from 28 countries, involving approximately 180 research centers. The United States (42 sites) and China (18 sites) contribute the largest number of research centers. At the macro level, China leads in total trial numbers (n=125), nearly double that of the United States (n=64), with Australia (n=23), Spain (n=22), and France (n=19) also showing significant activity. Despite the extensive international collaboration observed in BsAbs trials for CRC, our analysis reveals a striking underrepresentation of low- and middle-income countries (LMICs). Among the phase II/III trials, only one trial (TrialTroveID-550131, amivantamab in EGFR/c-MET-positive CRC) explicitly included an LMIC (India) as a participating region (**Supplementary Table 1**).

### 3.5 Landscape of bispecific antibody target combinations

Analysis reveals that PD-1-based pairs, particularly with CTLA-4 or VEGF, are the most prevalent strategies in bispecific antibody (BsAb) development for colorectal cancer (CRC), as indicated by the largest circle sizes representing trial frequency(**Figure 2E**). Clinical investigation of the PD-1/CTLA-4 axis is exemplified by the Phase II trial of cadonilimab (AK104) in metastatic CRC (NCT05747716). While some combinations, like PD-1/VEGF, are being explored in broader solid tumor trials due to shared oncogenic pathways, their investigation provides a critical rationale for CRC-specific applications, given the significant expression of these targets in CRC. Beyond these established immune-checkpoint combinations, emerging targets like EGFR/cMET are gaining traction. BsAbs such as amivantamab (JNJ-61186372), initially studied in broader solid tumors (NCT04606381), are highly relevant for CRC due to the critical role of cMET signaling in resistance to anti-EGFR therapy.

### 3.6 Comprehensive analysis of clinical efficacy data

In addition to the macro-analysis of trial volume, phase, and target distribution, this review also systematically compiles published or preliminarily disclosed efficacy data from relevant clinical trials (**Tables 1** and **2**). In the neoadjuvant treatment of locally advanced colorectal cancer, Cadonilimab demonstrated a high pathological complete response (pCR) rate (84.6%) and a 100% major pathological response (MPR) in dMMR/MSI-H patients. In pMMR/MSS patients, Cadonilimab combined with chemotherapy also showed superior pathological response and downstaging rates compared with chemotherapy alone. In systemic treatment for metastatic colorectal cancer, several BsAbs combined with chemotherapy or antiangiogenic agents have shown encouraging objective response rates (ORR) in MSS-type patients. For example, Cadonilimab combined with FOLFOXIRI and bevacizumab achieved an ORR of 100% in the first-line setting, and Ivonescimab combined with chemotherapy yielded an ORR exceeding 80%. Furthermore, Amivantamab targeting the EGFR/cMET pathway still achieved an ORR of 49% in treatment-refractory RAS/BRAF wild-type patients who had failed prior anti-EGFR therapy.

**Table 1:** Summary of efficacy from clinical trials of novel agents in neoadjuvant treatment for locally advanced colorectal cancer.

Bispecific antibody	Study design	Patient population	Treatment regimen	pCR rate	MPR/downstaging rate	Key safety profile
Cadonilimab	Single-arm Phase II (TrialTroveID-466260)	dMMR/MSI-H LACRC	Cadonilimab	84.6% (11/13)	MPR: 100% (13/13) R0 resection: 100% (13/13)	Grade 3–4 TRAEs: 14.7% (including myocarditis, myositis)
Cadonilimab	Randomized Phase II (TrialTroveID-444582)	pMMR/MSS LACRC	A:mFOLFOXIRI + Cadonilimab B:mFOLFOXIRI C:mFOLFOX6	A:26.8% B:15.4% C:9.8%	A: MPR 68.3%; Downstaging 65.9% B: MPR 48.7%; Downstaging 46.2% C: MPR 43.9%; Downstaging 41.5%	Not reported in detail
Cadonilimab	Single-arm Phase II (TrialTroveID-464109)	pMMR/MSS LARC	SCRT → Cadonilimab + mFOLFOX6	37.0% (10/27)	MPR: 55.6% T-downstaging: 59.3%; N-downstaging: 66.7%	Grade 3–5 AEs: 18.5%

AE, adverse event; dMMR, deficient mismatch repair; LACRC, locally advanced colorectal cancer; LARC, locally advanced rectal cancer; MPR, major pathological response; MSI-H, microsatellite instability-high; MSS, microsatellite stable; pCR, pathological complete response; pMMR, proficient mismatch repair; SCRT, short-course radiotherapy; TRAE, treatment-related adverse event.

**Table 2:** Summary of efficacy from clinical trials of novel agents in systemic treatment for metastatic colorectal cancer.

Bispecific antibody	Study design	Patient population & line of therapy	Treatment regimen	ORR	DCR/survival	Key safety profile
Cadonilimab	Single-arm Phase II (TrialTroveID-468024)	pMMR/MSS mCRC (1L)	Cadonilimab + Bevacizumab + FOLFOXIRI	100% (15/15)	DCR: 100% mPFS/mOS: Immature	Grade 3–4 AEs: Neutropenia (55%)
Ivonescimab	Randomized Phase II (TrialTroveID-432498)	MSS mCRC (1L) >50% KRAS/BRAF mutant	A:Ivonescimab + FOLFOXIRI B:Ivonescimab +Ligufalimab+ FOLFOXIRI	A:81.8% B:88.2%	DCR:100% (both groups) 9-month PFS Rate:A: 81.4%, B: 86.2%	Grade ≥3 TRAEs: A: 54.5%, B: 44.4%.(including neutropenia, leukopenia, diarrhea)
Amivantamab	Single-arm Phase I/II (TrialTroveID-432461)	RAS/BRAF WT, Anti-EGFR mCRC (1L/2L)	Amivantamab+ FOLFOX/FOLFIRI	49%	DCR: 88% mPFS:7.5 months mDoR:7.4 months	Neutropenia, rash. No additive toxicity
IBI363	Multi-cohort Phase I (TrialTroveID-547031)	MSS/pMMR mCRC (≥3L, Later-line)	Cohort 1:IBI363 Cohort 2:IBI363 + Bevacizumab	Cohort 1:12.7% Cohort 2:23.5%	Cohort 1:mOS 16.1 months Cohort 2:DCR 83.9% mPFS 9.6 months	Grade ≥3 TRAEs: Cohort 1: 23.5%, Cohort 2: 30.1%

1L, first-line; 2L, second-line; 3L, third-line; AE, adverse event; DCR, disease control rate;mCRC, metastatic colorectal cancer; mDoR, median duration of response; mOS, median overall survival; mPFS, median progression-free survival;ORR, objective response rate; PFS, progression-free survival; TRAE, treatment-related adverse event;

## 4 Discussion

From 2011 to 2025, the clinical trial field of bispecific antibodies (BsAb) for colorectal cancer (CRC) has undergone significant expansion, evolving from sporadic early explorations to a powerful drug development team with 192 studies. This progress is marked by a shift from the dominance of Phase I clinical trials to a surge in Phase II and III clinical trials starting in 2023-2024. The primary focus of these trials remains safety, and determining dose-limiting toxicity and the maximum tolerated dose (MTD) has become the top priority. The development of this field is mainly driven by the industrial sector, which sponsors 68.3% of clinical trials, while academic institutions contribute 28.5%. In addition, the treatment focus has been concentrated on dual blocking based on PD-1 and strategies targeting EGFR/cMET.

The prevalence of PD-1 combination therapy, especially PD-1/CTLA-4 and PD-1/VEGF combination therapy, aims to overcome the limitations of single-agent immunotherapy. Most colorectal cancers are microsatellite stable type (MSS), with a cold tumor immune microenvironment, and are insensitive to single treatment mainly based on immune checkpoint inhibitors (20). In the past, the main treatment methods were immune checkpoint inhibitors combined with chemotherapy or targeted therapy (21). In the neoadjuvant treatment of locally advanced colorectal cancer, cadonilimab, targeting PD-1/CTLA-4, has demonstrated a remarkably high pathological pCR rate (84.6%) and a 100% MPR rate in patients with dMMR/MSI-H tumors (TrialTroveID-466260). More importantly, in the pMMR/MSS population, cadonilimab combined with chemotherapy (mFOLFOXIRI) significantly increased both the pCR rate (26.8% *vs*. 9.8%) and the MPR/downstaging rate compared with chemotherapy alone (TrialTroveID-444582). These findings indicate that simultaneously blocking non-redundant immune checkpoints represents an emerging strategy—based on the principle of enhancing T-cell infiltration and activation—to convert these immunologically cold tumors into hot ones (22). Furthermore, the emergence of EGFR/cMET targeted bispecific antibodies (amivantamab) has addressed a key issue that urgently needs to be solved: acquired drug resistance (23). As cMET amplification is a known resistance mechanism to anti-EGFR therapies (cetuximab or panitumab), this dual-target drug provides mechanism rationality for reducing drug resistance in patients with MSS of CRC.

Unlike CAR-T cell therapy or TCR-engineered T cells, which achieve deep remissions in hematologic malignancies but remain constrained by limited trafficking and antigen loss in solid CRC (24, 25), BsAbs offer off-the-shelf availability and rapid T-cell redirection without ex vivo manipulation. Similarly, third-generation Antibody drug conjugate (26) provide potent bystander cytotoxicity yet rely on adequate target internalization and stable linker chemistry; dose-limiting interstitial lung disease has been reported with HER2-directed ADCs. In MSS-CRC, where tumor heterogeneity and low immunogenicity predominate, BsAbs therefore occupy a complementary niche—functioning as micro-environmental “igniters” that can be sequenced or combined with CAR-T/TCR-T depth or ADC payload, rather than competing head-to-head on single-agent efficacy.

The primary endpoints of most clinical trials, namely safety and tolerability, underscore the dual nature of BsAbs therapy: despite their potent antitumor efficacy, significant toxicities remain a serious concern. For example, in locally advanced disease, cadonilimab (TrialTroveID-466260)—either as monotherapy or in combination—has been associated with grade 3–4 treatment-related adverse events (TRAEs) including immune-related toxicities such as myocarditis and myositis. In metastatic settings, regimens combining BsAbs with chemotherapy frequently lead to a high incidence of hematological toxicities like neutropenia, with ≥ grade 3 events occurring in 44.4% to 55% of patients. Although T-cell redirection—an MHC-independent mechanism—delivers effective tumor killing, it also introduces risks such as on-target/off-tumor toxicity and cytokine release syndrome (CRS) (27). Extensive concern over the maximum tolerated dose (MTD) and dose-limiting toxicity indicates that the therapeutic indices of many such drugs are still being determined. Unlike various broad-spectrum chemotherapy drugs, bispecific antibodies require precise administration to balance immune activation and systemic safety. As the Phase II clinical trials determined for 2024–2025 are gradually launched, we expect endpoint priority to shift towards efficacy metrics such as overall survival (OS) and progression-free survival (PFS), which will ultimately determine their clinical application value.

The design and development of novel bispecific antibodies (BsAbs) for colorectal cancer are increasingly focusing on multi-target and multi-functionality to overcome tumor heterogeneity and therapeutic limitations. Innovative technologies such as trispecific T-cell adaptors (TriTEs), which can simultaneously target two antigens (such as EGFR and EpCAM), have demonstrated the ability to prevent antigen escape (15). Meanwhile, advanced delivery systems such as hollow mesoporous ruthenium nanoparticles and mrNA-lipid nanoparticles (LNP) platforms are being used to enhance tumor penetration and achieve *in situ* drug production (28, 29). In addition, artificial intelligence (AI) is being utilized to optimize structural affinity - for instance, by reducing the affinity of the CD3 domain - in order to balance potent cytotoxicity and lower the risk of cytokine release syndrome (30). Identifying predictive biomarkers is crucial for individualized treatment. The expression levels of CEA, EpCAM and LY6G6D are related to the efficacy of bispecific antibodies (BsAb) (16). Higher antigen density usually leads to enhanced cytotoxicity of tumor cells, and non-invasive plasma exosome markers (such as c-MET/PD-L1) are key indicators for predicting therapeutic effects (9).
