# Supplementary material for: Clinical trials of bispecific antibody therapy for colorectal cancer: advanced and next steps
Source: Front Oncol. 2026 Feb 11;16:1758251. doi: 10.3389/fonc.2026.1758251 (PMC12932179; doi:10.3389/fonc.2026.1758251)
Supplement: Supplementary file 1 [file Table1.docx]

****Supplementary Materials****

**Supplementary Table 1:** **Clinical characteristics of promising BsAbs in CRC phase II/III clinical trials**

| Trial ID | Bispecific Antibody | Targets | Phases | Trial Status | Sponsor Type | "Low income" or "lower middle income" countries | Start Year |
| --- | --- | --- | --- | --- | --- | --- | --- |
| TrialTroveID-208995 | vanucizumab | Ang2,VEGF | II | Terminated | Industry | No | 2014 |
| TrialTroveID-314375 | dilpacimab | DLL4,VEGF | II | Terminated | Industry | No | 2018 |
| TrialTroveID-334082 | BI-836880 | Ang2,VEGF | II | Completed | Industry | No | 2019 |
| TrialTroveID-327577 | zanidatamab | ECD2,ECD4 | II | Closed | Industry | No | 2019 |
| TrialTroveID-384964 | cadonilimab | PD-1,CTLA-4 | II | Planned | Academic | No | 2020 |
| TrialTroveID-382419 | KN-046  KN-026 | PD-L1,CTLA-4  ECD2,ECD4 | II | Completed | Industry  Academic | No | 2020 |
| TrialTroveID-402403 | SHR-1701 | PD-L1,TGF-βRII | II/III | Terminated | Industry | No | 2021 |
| TrialTroveID-413512 | izalontamab | EGFR,HER3 | II | Terminated | Industry | No | 2021 |
| TrialTroveID-371784 | zanidatamab | ECD2,ECD4 | II | Terminated | Industry | No | 2021 |
| TrialTroveID-432498 | ivonescimab | PD-1,VEGF | II | Open | Industry | No | 2022 |
| TrialTroveID-428008 | SHR-1701 | PD-L1,TGF-βRII | II | Open | Industry | No | 2022 |
| TrialTroveID-436805 | navicixizumab | DLL4,VEGF | II | Open | Industry | No | 2022 |
| TrialTroveID-430141 | vudalimab | PD-1,CTLA-4 | II | Temporarily Closed | Academic | No | 2022 |
| TrialTroveID-440496 | CTX-009 | DLL4,VEGF | II | Completed | Industry, | No | 2022 |
| TrialTroveID-460435 | cadonilimab | PD-1,CTLA-4 | II | Planned | Academic | No | 2023 |
| TrialTroveID-466261 | cadonilimab | PD-1,CTLA-4 | II | Open | Academic | No | 2023 |
| TrialTroveID-466260 | cadonilimab | PD-1,CTLA-4 | II | Open | Academic | No | 2023 |
| TrialTroveID-455531 | cadonilimab | PD-1,CTLA-4 | II | Open | Government Academic Industry | No | 2023 |
| TrialTroveID-464109 | cadonilimab | PD-1,CTLA-4 | II | Completed | Academic | No | 2023 |
| TrialTroveID-557877 | KN-046 | PD-L1,CTLA-4 | II | Terminated | Industry | No | 2023 |
| TrialTroveID-469230 | cadonilimab | PD-1,CTLA-4 | II | Open | Government | No | 2023 |
| TrialTroveID-473462 | vudalimab | PD-1,CTLA-4 | II | Terminated | Academic Industry | No | 2023 |
| TrialTroveID-495913 | cadonilimab | PD-1,CTLA-4 | II | Open | Academic | No | 2023 |
| TrialTroveID-474713 | cadonilimab | PD-1,CTLA-4 | II | Planned | Academic | No | 2023 |
| TrialTroveID-474362 | SHR-1701 | PD-L1,TGF-βRII | II | Planned | Academic | No | 2023 |
| TrialTroveID-485212 | cadonilimab | PD-1,CTLA-4 | II | Open | Academic Industry | No | 2023 |
| TrialTroveID-444582 | cadonilimab | PD-1,CTLA-4 | II | Completed | Academic | No | 2023 |
| TrialTroveID-481288 | KN-026 KN-046 | ECD2,ECD4 PD-L1,CTLA-4 | II | Planned | Academic | No | 2023 |
| TrialTroveID-472492 | volrustomig | PD-1,CTLA-4 | II | Planned | Academic Industry  Government | No | 2023 |
| TrialTroveID-480502 | cadonilimab | PD-1,CTLA-4 | II | Open | Academic Industry | No | 2023 |
| TrialTroveID-489916 | KN-046 | PD-L1,CTLA-4 | II | Open | Academic | No | 2023 |
| TrialTroveID-481276 | KN-046 | PD-L1,CTLA-4 | II | Open | Academic | No | 2023 |
| TrialTroveID-494546 | cadonilimab | PD-1,CTLA-4 | II | Open | Government | No | 2023 |
| TrialTroveID-482546 | SI-B003 | PD-1,CTLA-4 | II | Open | Industry | No | 2023 |
| TrialTroveID-468024 | cadonilimab | PD-1,CTLA-4 | II | Open | Academic | No | 2023 |
| TrialTroveID-494129 | volrustomig | PD-1,CTLA-4 | II | Planned | Academic | No | 2023 |
| TrialTroveID-501202 | cadonilimab | PD-1,CTLA-4 | II | Open | Academic | No | 2024 |
| TrialTroveID-532449 | cadonilimab | PD-1,CTLA-4 | III | Open | Academic | No | 2024 |
| TrialTroveID-505886 | IBI-363 | PD-1,IL-2α | II | Open | Industry | No | 2024 |
| TrialTroveID-521176 | cadonilimab | PD-1,CTLA-4 | II | Open | Academic | No | 2024 |
| TrialTroveID-527410 | cadonilimab | PD-1,CTLA-4 | II | Planned | Academic | No | 2024 |
| TrialTroveID-530731 | cadonilimab | PD-1,CTLA-4 | II | Open | Academic | No | 2024 |
| TrialTroveID-569628 | ivonescimab | PD-1,VEGF | II | Open | Academic | No | 2024 |
| TrialTroveID-525613 | SSGJ-707 | PD-1,VEGF | II | Open | Industry | No | 2024 |
| TrialTroveID-555220 | ivonescimab | PD-1,VEGF | II | Open | Academic | No | 2024 |
| TrialTroveID-542040 | amivantamab | EGFR,c-MET | III | Open | Industry | No | 2024 |
| TrialTroveID-540810 | cadonilimab | PD-1,CTLA-4 | II | Open | Academic | No | 2024 |
| TrialTroveID-536002 | ivonescimab | PD-1,VEGF | II | Planned | Academic | No | 2024 |
| TrialTroveID-552591 | ivonescimab | PD-1,VEGF | II | Planned | Academic | No | 2024 |
| TrialTroveID-543364 | SHR-1701 | PD-L1,TGF-βRII | II | Planned | Academic | No | 2024 |
| TrialTroveID-550131 | amivantamab | EGFR,c-MET | III | Open | Industry | Yes | 2024 |
| TrialTroveID-549722 | ivonescimab | PD-1,VEGF | II | Planned | Academic | No | 2024 |
| TrialTroveID-549606 | ivonescimab | PD-1,VEGF | II | Planned | Academic Industry | No | 2024 |
| TrialTroveID-544803 | zanidatamab | ECD2,ECD4 | II | Open | Industry | No | 2025 |
| TrialTroveID-551449 | ivonescimab | PD-1,VEGF | II | Planned | Academic | No | 2025 |
| TrialTroveID-547046 | ivonescimab | PD-1,VEGF | II | Open | Academic | No | 2025 |
| TrialTroveID-558186 | AP-505 | PD-L1,VEGF | II | Planned | Industry  Academic | No | 2025 |
| TrialTroveID-570859 | ivonescimab | PD-1,VEGF | II | Open | Academic | No | 2025 |
| TrialTroveID-559275 | ivonescimab | PD-1,VEGF | II | Open | Government | No | 2025 |
| TrialTroveID-557720 | cadonilimab | PD-1,CTLA-4 | III | Planned | Academic | No | 2025 |
| TrialTroveID-568168 | ivonescimab | PD-1,VEGF | II | Planned | Academic Industry | No | 2025 |
| TrialTroveID-562260 | JS-207 | PD-1,VEGF | II | Open | Industry | No | 2025 |
| TrialTroveID-554033 | ivonescimab | PD-1,VEGF | II | Planned | Academic | No | 2025 |
| TrialTroveID-559942 | amivantamab | EGFR,c-MET | II | Planned | Academic | No | 2025 |
| TrialTroveID-573210 | ivonescimab | PD-1,VEGF | III | Planned | Industry | No | 2025 |
| TrialTroveID-580717 | cadonilimab | PD-1,CTLA-4 | II | Open | Academic | No | 2025 |
| TrialTroveID-576372 | ivonescimab | PD-1,VEGF | II | Planned | Academic | No | 2025 |
| TrialTroveID-554451 | volrustomig | PD-1,CTLA-4 | II | Open | Industry | No | 2025 |
| NCT06724263 | B1962 | PD-L1,VEGF | II | Planned | Industry | No | 2024 |
| NCT07068763 | cadonilimab | PD-1,CTLA-4 | II | Planned | Other | No | 2025 |
